# P253: The infection surveillance related to care through the creation of repeated prevalence surveys

**DOI:** 10.1186/2047-2994-2-S1-P253

**Published:** 2013-06-20

**Authors:** H Kammoun, ML Atif

**Affiliations:** 1 Regional Directorate of Health Bizerte, Bizerte, Tunisia; 2 University Hospital of Blida, Blida, Tunisia

## Introduction

One easy method of monitoring healthcare associated infections is the conduct of repetitive prevalence surveys thus allowing to sensibilize a large number of professionals, to identify the priority issues of the prevention policy and to implement and assess the overall impact over time of a prevention policy. The difficulty of these investigations is the great variability in the population of patients from one period to the other people with regard to patient characteristics that may affect infection rates such as exposure to invasive procedures.

## Objectives

Here we propose a method of adjustment of prevalence rates based on patient characteristics and exposure to invasive procedures so that these rates are comparable over time: standardization.

## Methods

This method of adjustment was performed to compare over time the results of prevalence surveys conducted in the region of Bizerte (northern Tunisia) for seven years (2005-2011) at two regional hospitals 691 beds comprising 17 hospital services.

## Results

The descriptive analysis of the data collected during the seven years showed a significant reduction in the prevalence of the monitoring period (p = 0.03). Prevalence declined between 2005 and 2007. It rose from 7.4% in 2005 to 6.4% in 2007 to increase in 2008 to 11.5% (probably related to the change of the monitoring period). The rate then declined from 2009 (5.4%) to 4.8% in 2011. The analysis of the results by performing a standardization of variables on the risk factors of patients showed a significant difference in standardized prevalence rates over time (p = 0.006).

## Conclusion

These results could reflect the effectiveness of preventive measures implemented since 2005. The decrease in the prevalence of healthcare associated infections in our region should motivate teams to continue their efforts hygiene and improving the quality of care.

## Competing interests

None declared

